# Biweekly versus triweekly hydrogen peroxide shock treatment for controlling dental unit waterline contamination: a single-center pilot randomized clinical study

**DOI:** 10.3389/froh.2026.1842649

**Published:** 2026-09-30

**Authors:** Jingcheng Wen, Wenjing Liu, Tianyi Shao, Jing Chang, Yanying Li, Ting Shuai, Xiue Li

**Affiliations:** 1 Dental Clinic office, Peking University School and Hospital of Stomatology & National Center for Stomatology & National Clinical Research Center for Oral Diseases & National Engineering Research Center of Oral Biomaterials and Digital Medical Devices, Beijing, China; 2 Nursing Department, Beijing Stomatological Hospital, Capital Medical University, Beijing, China; 3 Fifth Clinical Division, Peking University School and Hospital of Stomatology & National Center for Stomatology & National Clinical Research Center for Oral Diseases & National Engineering Research Center of Oral Biomaterials and Digital Medical Devices, Beijing, China; 4 Third Clinical Division, Peking University School and Hospital of Stomatology & National Center for Stomatology & National Clinical Research Center for Oral Diseases & National Engineering Research Center of Oral Biomaterials and Digital Medical Devices, Beijing, China; 5 Second Clinical Division, Peking University School and Hospital of Stomatology & National Center for Stomatology & National Clinical Research Center for Oral Diseases & National Engineering Research Center of Oral Biomaterials and Digital Medical Devices, Beijing, China

**Keywords:** biofilm, dental unit waterlines, hydrogen peroxide, shock treatment, water quality

## Abstract

**Background:**

This single-center pilot randomized clinical study compared the efficacy of biweekly and triweekly 1.41% hydrogen peroxide (H_2_O_2_) shock treatments, combined with continuous 0.0141% H_2_O_2_ disinfection, for controlling microbial contamination and biofilm formation in dental unit waterlines (DUWLs).

**Methods:**

Eight dental chair units (DCUs) were randomized to Group A (triweekly shock treatment, *n* = 4) or Group B (biweekly shock treatment, *n* = 4). After standardized baseline biofilm removal using BRS®, total viable counts (TVCs) and biofilm architecture assessed qualitatively by scanning electron microscopy (SEM), were monitored for 6 weeks. One randomly selected DCU (group A1 and B1) from each group underwent an exploratory additional 6-week observation, with the resulting data analyzed descriptively.

**Results:**

Group B maintained a 100% compliance rate (≤ 100 CFU/mL) throughout the 6-week period, whereas Group A showed a significantly lower compliance rate of 78.47%, with recurrent microbial rebound during the third week of each cycle. Biweekly shock treatment increased the probability of compliance by 21.53 percentage points compared with triweekly treatment (95% CI: 13.74–29.32, Wald *χ^2^* = 29.34, *P* < 0.001). Significant between-group differences in TVCs emerged by day 17 (*P* < 0.05). During the 12-week observation, Group B1 remained compliant, whereas Group A1 showed recurrent non-compliant TVCs during the third week of several cycles. SEM observations showed biofilm-like structures in the sampled tubing segment from Group A after 6 weeks, whereas no obvious mature biofilm-like structure was observed in the sampled segment from Group B.

**Conclusions:**

Within the limitations of this single-center pilot study, continuous disinfection with 0.0141% H_2_O_2_ combined with biweekly 1.41% H_2_O_2_ shock treatment was associated with more consistent water-quality compliance than triweekly shock treatment after initial BRS® biofilm removal. These preliminary findings require confirmation in larger multicenter, year-round studies incorporating longer follow-up and microbial identification before any change to routine clinical protocols can be considered.

## Introduction

During dental treatment, both patients and oral healthcare providers are exposed to microbial risks from dental unit waterlines (DUWLs) via contact with contaminated water and inhalation of aerosols and splatter (1, 2). DUWLs consist of narrow plastic tubing supplying water to high-speed handpieces and air–water syringes, whose small diameter and complex configuration favour nutrient deposition, microbial adhesion, and biofilm formation (3, 4). Although the American Centers for Disease Control and Prevention (CDC) recommends that dental treatment water should contai*n* ≤ 500 colony-forming units per milliL (CFU/mL) (5), actual contamination levels frequently exceed this threshold (6–8), with detection of pathogens such as *Pseudomonas aeruginosa* and *Legionella spp* (9, 10)., thereby increasing the risk of healthcare-associated infections, particularly in immunocompromised patients (11, 12). Following the Coronavirus Disease 2019 (COVID-19) pandemic, concerns regarding airborne transmission in dental settings have further intensified (13). Reflecting this, the Chinese Stomatological Association (CSA) mandated a limit of ≤ 100 CFU/mL for dental treatment water in China (14).

Current DUWLs control strategies typically combine physical and chemical measures (15). Physical approaches (e.g., anti-retraction valves, routine flushing, and optimized tubing materials) can reduce microbial proliferation but cannot reliably remove established biofilms in narrow-bore lines (16–18). Consequently, chemical disinfectants such as sodium hypochlorite, hypochlorous acid, and hydrogen peroxide (H_2_O_2_) are widely used (19, 20). H_2_O_2_ offers broad-spectrum activity, rapid action, low toxicity, and good environmental compatibility, but its intrinsic instability limits long-term efficacy (21). Professional guidelines therefore advocate combining continuous low-level dosing with periodic high-concentration shock disinfection to achieve more sustained control of DUWLs contamination (22, 23). To improve the stability and performance of H_2_O_2_, compound formulations such as Dentosept® S have been developed. *In vitro*, continuous low-concentration Dentosept® S with the manufacturer-recommended four-week shock interval reduced biofilm bacterial counts ≤ 100 CFU/mL (3), and another study reported bacterial levels < 200 CFU/mL in over 91% of water samples (24). However, a clinical study in China using this standard regimen achieved only 89.9% compliance after 25 weeks, with bacterial counts regularly increasing during the third week of each cycle (25). These findings suggest that a four-week shock interval may be insufficient under domestic clinical conditions, and that the optimal shock frequency remains unclear.

To address this gap, our previous pilot study compared different shock intervals under continuous low-concentration H_2_O_2_ dosing (26). Weekly and biweekly H_2_O_2_ shock disinfection both achieved 100% water-quality compliance, whereas the triweekly regimen achieved only 68.42%. These preliminary findings suggested that shorter intervals may be more effective than the commonly used triweekly schedule. However, the pilot study had a limited sample size and short follow-up, which were insufficient to support an immediate change in routine practice. In addition, although weekly shock disinfection was effective, it is difficult to implement in busy dental clinics because of the added workload and cost. In real-world practice, the relevant unresolved question is therefore whether shortening the shock-treatment interval from three weeks to two weeks could improve water-quality compliance under routine clinical conditions. Accordingly, the present pilot study compared biweekly and triweekly H_2_O_2_ shock disinfection, combined with continuous low-concentration H_2_O_2_ dosing, to generate preliminary comparative clinical data on water-quality compliance in Chinese dental clinics.

## Materials and methods

### Dental chair unit selection

This study was conducted between May and August 2024 at a dental teaching hospital in China. The sample size was determined by the number of eligible DCUs available for the pilot study. Thus eight dental chair units (DCUs) (Intego Pro, Dentsply Sirona, USA) were selected as research subjects. Four units were from the Department of Prosthodontics and four were from the Department of General Dentistry. All had been in clinical use for 10 months to 4 years, had not undergone chemical disinfection previously, and had not had their DUWLs replaced. All DCUs were equipped with automatic flushing and disinfection systems, and filtered municipal water was used as the input source. To minimize confounding, the eight DCUs were allocated equally to Group A (*n* = 4) and Group B (*n* = 4) by department-stratified randomization. Within each departmental stratum, the four DCUs were allocated in a 1:1 ratio to Group A or Group B using a computer-generated random permutation containing two assignments to each group. Randomization was completed before implementation of the disinfection protocols. After the initial 6-week disinfection and evaluation phase, one unit from each group, designated Group A1 and Group B1, was followed for an additional 6 weeks of exploratory observation of total viable counts (TVCs), and these data were analyzed descriptively.

### Baseline biofilm removal protocol

Prior to the implementation of the disinfection protocols, a standardized biofilm removal procedure was performed using the Biofilm-Removing-Set (BRS®, ALPRO MEDICAL GMBH, Germany) to restore the DUWLs to a near-original state per ISO 16954-2015 (27) standard test method. The BRS® includes the BRS® PreCleaner, BRS® Remover, and BRS® Activator. On a Saturday afternoon, 10 g BRS® PreCleaner was dissolved in 2 L of 50 ℃ water and flushed through the lines using a vibrating piston pump for a period not exceeding 15 min. This was followed by a solution of 160 g BRS® Remover and 20 mL BRS® Activator dissolved in 2 L of 50-60 ℃ water, which was also flushed through the lines. Subsequently, 1 L of the blue solution Bilpron® was introduced into the built-in water tanks and allowed to dwell in the DUWLs for 24 h. On the following Sunday afternoon, the lines were flushed with municipal water until no blue color remained in the water, followed by an additional 30-second flush. Water samples were collected immediately after flushing to assess the total bacterial count, and if the count exceeded 100 CFU/mL, the biofilm removal protocol was repeated.

### Continuous disinfection protocol

During the study period, continuous low-concentration disinfection was performed using Dentosept® S (ALPRO MEDICAL GMBH, Germany), a H_2_O_2_-based disinfectant composed of 1.41% H_2_O_2_, 98.5% water, and less than 0.1% stabilizers. According to the manufacturer's instructions for continuous DUWLs disinfection, the DCU automatic disinfection system mixed municipal input water with the 1.41% H_2_O_2_ concentrate at a ratio of 100:1, yielding a constant treatment-water concentration of 0.0141% H_2_O_2_. Before study commencement, engineers tested the performance of all DCUs. This ensured that the automatic systems could accurately execute the commands for disinfectant dilution and flushing. In accordance with the guideline (14), the automatic system performed a 2-minute DUWL flush followed by a drying cycle at the start and end of each clinical session. In addition, to reduce the risk of cross-contamination, all high-speed handpieces were run idle for 30 s between patients.

### Shock disinfection protocol

In addition to continuous disinfection, periodic shock treatments with undiluted Dentosept® S were carried out every 3 weeks in Group A and every 2 weeks in Group B. At the end of clinical sessions on Friday afternoon, the DCU automatic disinfection system filled the DUWLs with 1.41% H_2_O_2_ solution for a 24-hour dwell period. On Saturday afternoon, the system automatically flushed the lines with municipal water and completed a drying cycle to prepare the units for subsequent clinical use. To ensure water safety, an additional shock disinfection cycle was performed before reuse if a DCU had remained idle for more than 3 days.

### Water sample collection

424 water samples were collected from the outlets of high-speed handpieces and air-water syringes across three study phases. Phase I established baseline levels and the efficacy of the BRS® biofilm removal procedure by sampling all eight DCUs for three consecutive days prior to the BRS® implementation and once immediately following the procedure. Phase II spanned a 6-week disinfection period, during which water samples were obtained from all eight units every Monday, Wednesday, and Friday morning before the start of clinical operations. Phase III involved an additional 6-week observation of two randomly selected units, representing one from Group A and one from Group B, designated Group A1 (*n* = 1) and Group B1 (*n* = 1), following the same sampling schedule.

For each sample, 10 mL of water was collected into a sterile tube after a 2-minute flush and surface disinfection of the outlet with a 75% ethanol swab. To ensure analytical accuracy, each sample was immediately mixed with 30% sodium thiosulfate solution at a ratio of 3:1 to neutralize residual H_2_O_2_.

### Tubing-segment collection for qualitative SEM assessment

To provide qualitative morphological information on DUWL surface deposits, tubing segments were excised from high-speed handpiece waterlines. Because SEM sampling required destructive tubing excision and the air-water syringe tubing in the clinical DCU had limited spare length, SEM sampling was restricted to high-speed handpiece tubing to avoid affecting subsequent clinical use. One DCU was randomly selected from Group A and Group B, respectively, and 1-cm segments were harvested using sterile scissors at three predetermined time points: prior to BRS® implementation, immediately following the BRS® procedure, and upon completion of the 6-week disinfection period. All segments were immediately transported to the laboratory for analysis. Samples that could not be processed immediately were maintained at 4 °C and analyzed within 24 h to preserve surface-associated material and morphology.

### Biofilm sample preparation and observation

Biofilm architecture on the internal surfaces of the high-speed handpiece tubing was examined using a JSM-5600 scanning electron microscope (SEM) (JEOL, Japan) as a qualitative assessment. To eliminate potential contamination, 0.1 cm was trimmed from each end of the 1-cm tubing segment with sterile scissors, and the segment was then slit longitudinally to expose the inner lumen. The specimens were fixed in 2.5% glutaraldehyde at room temperature for 1 h and dehydrated through a graded ethanol series (10%, 30%, 50%, 75%, 90%, and 100%) for 5 min at each concentration, with the 100% step repeated three times. After air-drying, the samples were mounted on SEM stubs with conductive adhesive and sputter-coated with gold to improve conductivity. The prepared inner surfaces were scanned and photographed to qualitatively document changes in surface-associated deposits and morphology.

### Determination of TVCs

TVCs were used to quantify the total burden of culturable bacteria in output water but did not identify microbial taxa or non-culturable organisms. 1 mL of the neutralized water sample was spread evenly in a sterile petri dish and mixed thoroughly with 20 mL of nutrient agar medium (Aoboxing Biotech Co., Beijing, China). After solidification, the plates were inverted and incubated at 36 ± 1 ℃ for 48 h. Colonies were then counted and recorded. According to the CSA guideline, dental treatment water was considered compliant when the TVC did not exceed 100 CFU/mL (14). The compliance rate was calculated as the number of compliant samples divided by the total number of samples collected in each group during a specific study phase.

### Blinding of sample collection and outcome assessment

Only the personnel responsible for implementing the scheduled shock disinfection were aware of group allocation. Sample collectors were blinded to the assigned disinfection interval, and all water and tubing samples were labeled with coded identifiers that did not disclose treatment allocation. Two laboratory assessors, both blinded to group allocation, performed and cross-checked TVC colony counting and qualitative SEM assessment using the coded identifiers. Any discrepancies were reviewed and resolved by consensus before the results were recorded.

### Statistical analysis

SPSS version 26.0 (Chicago, IL, USA) was used for descriptive and inferential analyses. Normally distributed variables are presented as mean ± standard deviation (SD), and between-group comparisons were performed using the independent-samples t test. Non-normally distributed data are presented as median (Q1, Q3), and between-group comparisons were performed using the Mann–Whitney U test. Categorical variables are presented as frequencies and percentages. Water-quality compliance was analyzed as the primary binary outcome. Generalized estimating equations (GEE) were used to compare compliance between groups while accounting for repeated measurements within DCUs. DCU was specified as the clustering unit, with an exchangeable working correlation structure and robust standard errors. Group was included as the main explanatory variable, and outlet site and sampling day were included as covariates. A two-sided *P* value < 0.05 was considered statistically significant.

## Results

### Baseline status and BRS® efficacy

Both Group A (*n* = 4) and Group B (*n* = 4) operated 5.5 working days weekly, with respective average weekly patient volumes of 226 and 223. Prior to the intervention, all DCUs demonstrated heavy microbial contamination. Before the BRS® procedure, TVCs at high-speed handpiece and air-water syringe outlets showed no statistically significant difference between groups (high-speed handpiece: Z = −0.289, *P* = 0.773; air-water syringe: Z = −0.866, *P* = 0.149).

After the BRS® procedure, the microbial load in all DUWLs decreased to compliant levels. No significant between-group differences were observed at either the high-speed handpiece or air-water syringe outlets (high-speed handpieces: Z = −1.461, *P* = 0.144; air-water syringes: Z = −0.289, *P* = 0.773). The highest bacterial count recorded across all units was 58 CFU/mL, indicating that the BRS® procedure effectively restored the waterlines to a near-original clean state before the disinfection phase (Table 1).

**Table 1:** Total viable counts in output water from DCUs [median (Q1, Q3)].

Sampling time	Group A (CFU/mL)		Group B (CFU/mL)		HSHP			AWS
	HSHP	AWS	HSHP	AWS	*Z*	*P*	*Z*	*P*
2 days before BRS®	9,815 (8575, 11,985)	9,185 (7772.5, 15,990)	8,435 (4402.5, 13,210)	6,315 (1790, 8740)	−0.289	0.773	−1.443	0.149
1 day before BRS®	8,800 (6220, 15,595)	6560 (2100, 25,480)	7880 (4827.5, 11,105)	8445 (3097.5, 8940)	−0.289	0.773	−0.577	0.564
On the morning of BRS®	11,460 (8162.5, 17,570)	7,100 (5,055, 19,585)	11,715 (9,555, 13,537.5)	9,670 (8570, 10882.5)	−0.289	0.773	−0.866	0.386
Immediately after BRS®	17.50 (12, 31.25)	19.5 (15.75, 36)	39.5 (20, 44.75)	22.5 (14.5, 50)	−1.461	0.144	−0.289	0.773
Day 1	23.5 (16.25, 33.75)	21.5 (18.75, 35.5)	35.5 (18.5, 45)	28.5 (18.25, 50)	−1.016	0.309	−0.436	0.663
Day 3	26.5 (11.75, 37.5)	26.5 (16.5, 36.5)	22.5 (16.5, 29.25)	25 (7.75, 27.25)	−0.289	0.773	−0.581	0.561
Day 5	31 (24.75, 38.75)	29.5 (18.5, 32.25)	24.5 (20.5, 30.75)	26 (19.75, 35.25)	−1.307	0.191	−0.145	0.885
Day 8	22.5 (10.25, 30.25)	20.5 (14.25, 31.25)	27.5 (17.75, 34.25)	39.5 (28.5, 58)	−0.726	0.468	−2.021	**0**.**043**
Day 10	25 (15.5, 27)	21.5 (20.25, 34.75)	25 (14.25, 56.75)	55 (27.5, 81.75)	0.000	1.000	−1.732	0.083
Day 12	25.5 (19.5, 43.5)	35 (29.25, 43)	43.5 (24.75, 57)	29.5 (25.75, 52.75)	−1.155	0.248	−0.577	0.564
Day 15	52 (18.75, 89)	51.5 (34.75, 79.5)	25.5 (19.75, 28.25)	18.5 (11.75, 23)	−0.289	0.773	−2.309	**0**.**021**
Day 17	55.5 (35, 226.75)	95.5 (55.25, 108.75)	22.5 (18.5, 38.5)	28.5 (25, 36.5)	−2.021	**0**.**043**	−2.309	**0**.**021**
Day 19	177 (46.25, 359.5)	129.5 (57.25, 210)	38.5 (19.5, 49.25)	38 (12.5, 59)	−1.732	0.083	−1.732	0.083
Day 22	19 (9, 23.75)	17.5 (13.25, 19.5)	36.5 (20.5, 61.5)	40 (34.75, 44.5)	−1.597	0.110	−2.323	**0**.**020**
Day 24	14.5 (12.5, 39)	22 (15.25, 33.25)	48.5 (35.25, 52)	35 (22.25, 65.75)	−2.021	**0**.**043**	−1.162	0.245
Day 26	38.5 (17.5, 67)	33 (21.25, 47.75)	60 (30, 71.25)	46.5 (43, 54.5)	−0.866	0.386	−1.607	0.108
Day 29	43 (35.75, 180.75)	43.5 (21.5, 96.25)	20 (15, 32.5)	18.5 (12, 40)	−2.021	**0**.**043**	−1.307	0.191
Day 31	72.5 (53.25, 424.75)	54.5 (41.5, 406.5)	23 (14.75, 58.25)	30.5 (13.5, 45.25)	−1.732	0.083	−1.732	0.083
Day 33	86.5 (59.25, 339.5)	77.5 (62.25, 321.5)	32.5 (23, 46.5)	39 (31.25, 43)	−2.309	**0**.**021**	−2.323	**0**.**020**
Day 36	224.5 (80.25, 470)	136 (76.25, 376.5)	27.5 (23.75, 59)	30.5 (18.75, 36.25)	−2.309	**0**.**021**	−2.309	**0**.**021**
Day 38	335 (217, 390)	248.5 (126.25, 280)	39.5 (25.25, 59.75)	32.5 (23.25, 41)	−2.309	**0**.**021**	−2.323	**0**.**020**
Day 40	340 (271.5, 537.5)	320 (157, 600)	39.5 (34.25, 59)	33 (22.5, 54)	−2.309	**0**.**021**	−2.309	**0**.**021**

CFU/mL, colony-forming units per milliliter; HSHP, high-speed handpieces; AWS, air-water syringe; Group A, underwent shock treatment every 3 weeks; Group B, underwent shock treatment every 2 weeks.

Bold values indicate statistically significant between-group differences (*P* < 0.05).

### 6-week disinfection efficacy

During the 6-week disinfection period, each group contributed 144 water samples, collected from four DCUs at two outlet sites over 18 sampling time points. Group B (biweekly shock treatment) achieved a 100% compliance rate (144/144), with TVCs in all high-speed handpieces and air-water syringe samples consistently remaining below 100 CFU/mL (Figure 1). In contrast, Group A (triweekly shock treatment) exhibited a lower overall compliance rate of 78.47% (113/144). Because repeated samples from the same DCUs were not independent, the primary compliance outcome was analyzed using a GEE-based linear probability model, with DCU specified as the clustering unit, an exchangeable working correlation structure, and robust standard errors. After adjustment for outlet site and sampling day, the difference in compliance between the two shock treatment intervals remained significant (Wald *χ^2^* = 29.34, *P* < 0.001). Compared with triweekly shock treatment, biweekly shock treatment increased the probability of compliance by 21.53 percentage points (95% CI: 13.74–29.32, *P* < 0.001). Notably, TVCs in Group A remained compliant during the first two weeks following a shock treatment but increased sharply during the third week of each cycle (Figure 1). Significant between-group differences in TVCs emerged as early as Day 17 (high-speed handpieces: Z = −2.021, *P* = 0.043; air-water syringe: Z = −2.309, *P* = 0.021). Specifically, air-water syringe samples showed significant differences at multiple intervals (*P* < 0.05), including Days 8, 15, 17, 22, 33, 36, 38, and 40. For high-speed handpieces samples, significant divergence was observed on Day 17, 24, 29, 33, 36, 38, and 40 (Table 1).

**Figure 1:**
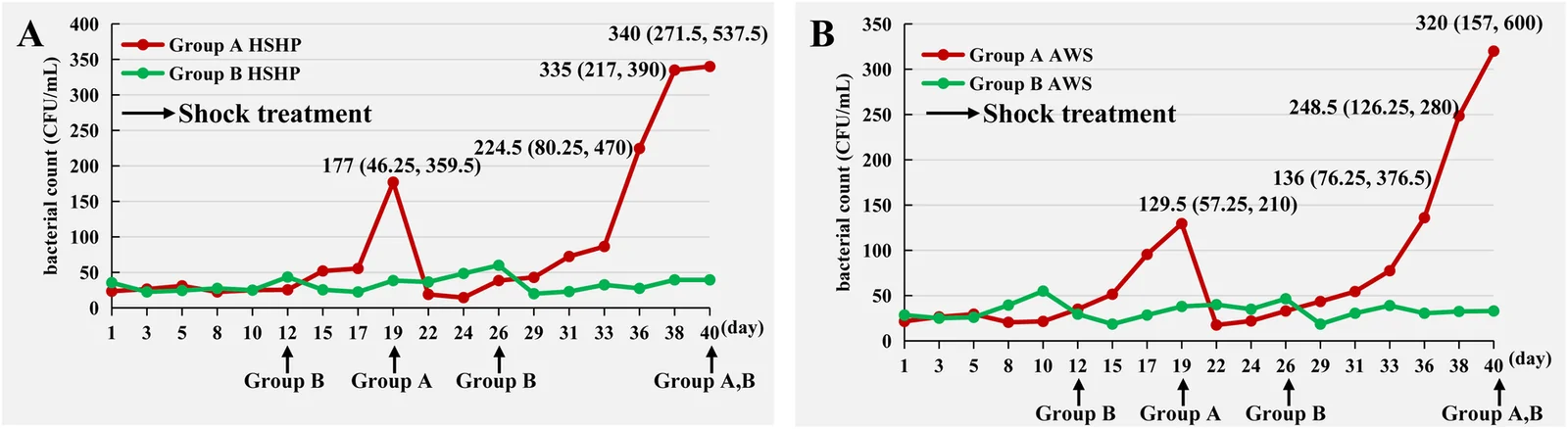
Trends in total viable counts in water samples from groups A and B during the 6-week study period. **(A)** total viable counts in water samples from the high-speed handpiece outlet; **(B)** total viable counts in water samples from the air-water syringe outlet.

#### Descriptive 12-week observations

The additional 6-week follow-up involved only one DCU from each group and was therefore analyzed descriptively. Over the 12-week period, Group B1 consistently maintained TVCs below 100 CFU/mL in all sampled water from both high-speed handpieces and air-water syringes (Figure 2). Notably, a gradual upward trend in TVC was observed during the final sampling period (Day 75–82) although all values remained compliant. In contrast, beginning with the second disinfection cycle (defined as one 3-week interval), Group A1 showed a recurrent increase in microbial counts during the third week of each cycle, with TVCs exceeding 100 CFU/mL. Specifically, TVCs surpassed the compliant limit between Days 29–40 during the second cycle, Days 59–61 during the third cycle and again around Days 80–82 during the fourth cycle. Interestingly, the maximum observed TVC values appeared lower in later cycles than in earlier cycles.

**Figure 2:**
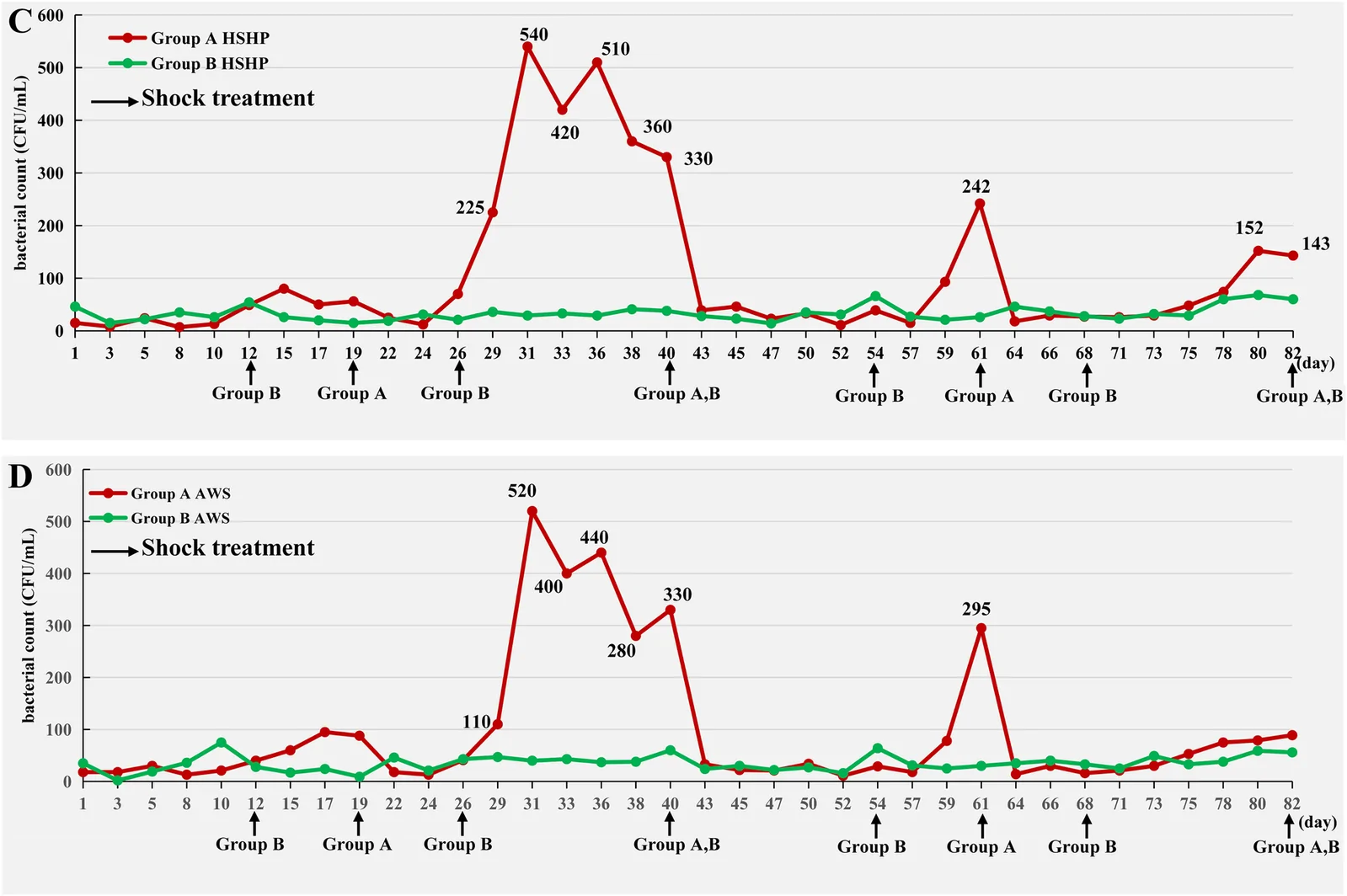
Trends in total viable counts in water samples from groups A1 and B1 during the 12-week observation period. **(C)** total viable counts in water samples from the high-speed handpiece outlet; **(D)** total viable counts in water samples from the air-water syringe outlet.

#### Qualitative SEM observations of high-speed handpiece tubing surfaces

Figure 3 shows SEM images of high-speed handpiece waterlines from randomly selected DCUs in Groups A and B at three time points: before BRS® treatment (Figures 3a,3d), immediately after the BRS® procedure (Figures 3b,3e), and after 6 weeks of disinfection (Figures 3c,3f). All images were captured at 5000x magnification in secondary electron mode with an acceleration voltage of 5.0 kV.

**Figure 3:**
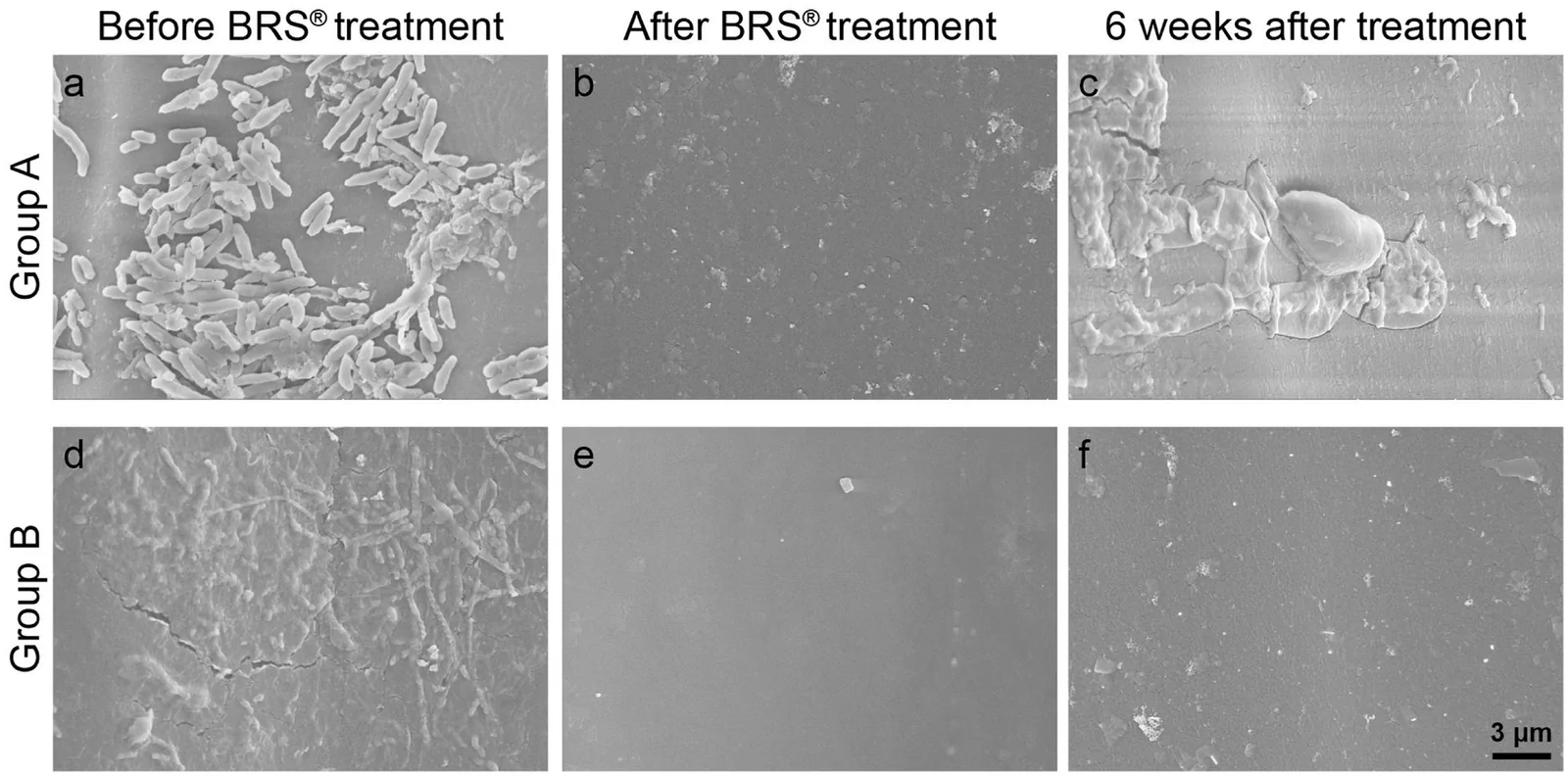
SEM images showing surface morphology in high-speed handpiece waterline tubing. **(a,d)** surface morphology before biofilm removal with BRS® and subsequent disinfection; **(b,e)** surface morphology following treatment with BRS®; **(c)** surface morphology after 6 weeks of treatment in Group A; **(f)** surface morphology after 6 weeks of treatment in Group B. Scale ba*r* = 3*μ*m.

Before any intervention, the inner tubing surfaces showed extensive surface-associated material. Dense aggregates of rod-shaped structures were tightly attached to the tubing surface (Figure 3a), and some structures appeared embedded within continuous matrix-like material (Figure 3d). These features were morphologically compatible with biofilm-like accumulation; however, their biological identity could not be confirmed by SEM alone. After the BRS® biofilm-removal procedure, the sampled tubing segments showed marked reduction in surface-associated material and cleaner inner surface as observed in SEM images (Figures 3b,3e).

After 6 weeks of disinfection, the sampled segment from Group A showed renewed accumulation of dense rod-shaped and matrix-like structures (Figure 3c). In contrast, no obvious dense biofilm-like structure was observed in the examined field from Group B, although scattered irregular particles were present (Figure 3f). Because standard SEM morphology alone cannot determine whether these particles were biological or non-biological, their biological nature could not be confirmed. These qualitative SEM findings were consistent with the differences in water-quality compliance observed between the two groups, but were not used to determine the biological nature of the observed particles.

## Discussion

This single-center pilot randomized clinical study showed that, after baseline biofilm removal with BRS®, a biweekly 1.41% H_2_O_2_ shock-disinfection schedule combined with continuous 0.0141% H_2_O_2_ treatment maintained 100% compliance with the CSA standard for DUWL water quality during the 6-week. By contrast, the triweekly schedule was associated with a lower compliance rate and recurrent TVC rebound during the third week of each cycle. These findings suggest that, after initial BRS® biofilm removal, shortening the maintenance shock-treatment interval from three weeks to two weeks may improve the consistency of DUWL water-quality compliance under the conditions of this single-center pilot study.

Our findings differ from those of Zemouri et al. (3), who suggested that a 4-week shock-disinfection interval might be adequate. Several factors may explain this discrepancy. First, our study was conducted in a real-world clinical setting rather than under *in vitro* conditions. As such, it captured practical factors that may promote recolonization, including handpiece backflow, intermittent water stagnation during shutdown periods, and variation in clinical workload. Second, we used the stricter CSA microbiological threshold of ≤ 100 CFU/mL rather than the CDC benchmark of < 500 CFU/mL. Third, the Dentosept® S formulation used in this study is silver-ion-free, which may have reduced its residual antimicrobial effect and thereby increased the need for more frequent shock treatment (28, 29). Taken together, these factors may explain why the manufacturer-recommended interval appeared insufficient in our clinical context.

The present study extends our preliminary pilot work (26) by using a randomized design, including a larger number of dental chair units, and involving both prosthodontic and general dentistry departments. These design features strengthen the clinical relevance of the findings. In particular, evidence from sequencing-based studies suggests that microbial communities in DUWLs may vary across dental specialties (30), which may in turn influence the effectiveness of decontamination strategies. The consistency between the pilot study and the present trial therefore provides more preliminary support for further evaluation of a biweekly shock-disinfection interval in larger clinical studies.

An important finding of this study was the recurrent third-week increase in TVCs observed in the triweekly group during the 6-week. This pattern was consistent with the report by Kramer et al. (31) and suggests that, under the conditions of this pilot study, the third week after shock treatment may represent a vulnerable period for loss of TVC-based water-quality compliance. In parallel, qualitative SEM observations showed renewed accumulation of dense rod-shaped and matrix-like structures in the sampled segment from the selected triweekly DCU (Group A) after 6 weeks, whereas no obvious mature biofilm-like structure was observed in the examined field from the selected biweekly DCU (Group B). These observations were consistent with the recurrent TVC rebound in Group A. However, because SEM cannot distinguish biological particles from non-biological deposits, these images should be interpreted as qualitative morphological evidence rather than direct proof of biofilm regeneration. The descriptive 12-week extension also showed recurrent third-week non-compliance in Group A1, but this observation was based on only one DCU per group and should be regarded as hypothesis-generating. The apparent decline in rebound peaks over successive cycles in Group A1 may reflect unit-specific variation, stochastic fluctuation, or changes in residual culturable bacterial burden under repeated shock treatment, and requires confirmation in larger longitudinal studies. From the perspective of hospital infection prevention and control, although the biweekly regimen was associated with lower TVCs and more consistent compliance with the CSA threshold, these findings cannot be interpreted as evidence that opportunistic pathogens were absent or reduced. The clinical significance of the observed differences in TVCs therefore requires further validation using pathogen-specific testing and molecular microbiological analyses.

Another noteworthy aspect of the study is the use of BRS® pretreatment to standardize the baseline condition of the DUWLs. Previous studies have shown that contamination in DUWLs may develop rapidly after commissioning and that once established, biofilms can be difficult to eradicate completely (32–35). Without baseline standardization, residual biofilm burden could confound comparisons between maintenance protocols. In our study, qualitative SEM observations showed a marked reduction in surface-associated biofilm-like material after BRS® pretreatment, consistent with previous reports (36). This methodological step improves confidence that the observed between-group differences were less likely to be explained by unequal initial contamination levels. However, it also limits the scope of interpretation. Our findings apply primarily to DUWLs after initial biofilm removal and baseline standardization. They should not be generalized directly to DUWLs that have not undergone baseline biofilm removal or to units with persistent established biofilm.

These pilot findings provide preliminary clinical evidence to guide the design of larger studies evaluating optimized DUWL shock treatment intervals, particularly when stringent water-quality standards are applied. After initial BRS® biofilm removal, a biweekly shock-disinfection schedule combined with continuous low-level H_2_O_2_ treatment was associated with more consistent compliance in busy dental settings. Nevertheless, the biweekly regimen should be considered a candidate strategy requiring further validation rather than a definitive replacement for existing protocols. Future implementation studies should evaluate workload, disinfectant consumption, feasibility, material compatibility, and long-term sustainability in routine practice.

Several limitations should be acknowledged. First, this single-center pilot study involved a limited number of DCUs, which restricts the statistical power and generalizability of the findings. Data were collected in Beijing from May to August 2024, and DUWL contamination and H₂O₂ performance may vary with source water quality, water hardness, residual municipal disinfectants, ambient temperature, DUWL design, patient volume, and local maintenance protocols. Second, the extended 12-week observation included only one randomly selected DCU per group and was therefore descriptive rather than representative of group-level long-term performance. Third, the study relied on TVCs and qualitative SEM observations. TVCs quantified only culturable bacterial burden and could not identify opportunistic pathogens, detect viable-but-nonculturable organisms, or characterize microbial community changes. SEM provided only qualitative morphological information from limited high-speed handpiece tubing segments and could not quantify biofilm biomass, determine biofilm dynamics, or distinguish biological from non-biological particles. Future studies should include larger multicenter sampling, longer follow-up, paired biofilm assessment of both outlet types, microbial identification, pathogen-targeted qPCR, 16S rRNA sequencing, quantitative biofilm assays, and implementation evaluations of cost, workload, material compatibility, and long-term sustainability.

## Conclusions

Within the limitations of this single-center pilot study, continuous disinfection with 0.0141% H_2_O_2_ combined with biweekly 1.41% H_2_O_2_ shock treatment was associated with more consistent compliance with the CSA TVC threshold than triweekly shock treatment after initial BRS® biofilm removal. These preliminary findings suggest that the biweekly regimen is a candidate interval for further evaluation, but they are not sufficient to support a definitive change in routine clinical protocols. Larger multicenter, year-round studies incorporating longer follow-up, microbial identification, microbial community analysis, and quantitative biofilm assessment are needed.

## Data Availability

The original contributions presented in the study are included in the article/Supplementary Material, further inquiries can be directed to the corresponding authors.

## References

[1] Hamilton KA Kuppravalli A Heida A Joshi S Haas CN Verhougstraete M. Legionnaires' Disease in dental offices: quantifying aerosol risks to dental workers and patients. J Occup Environ Hyg. (2021) 18(8):378–93. 10.1080/15459624.2021.1939878 34161202

[2] Allison JR Dowson C Jakubovics NS Nile C Durham J Holliday R. Waterline disinfectants reduce dental bioaerosols: a multitracer validation. J Dent Res. (2022) 101:1198–204. 10.1177/00220345221093522 35492016 PMC9397394

[3] Zemouri C De Soet JJ Volgenant CMC Crielaard W Laheij AMGA. Heterogeneity in the efficacy of dental chemical disinfectants on water-derived biofilms *in vitro*. Biofouling. (2020) 36(5):587–96. 10.1080/08927014.2020.1782894 32580580

[4] Kader CB De Smidt O Oosthuysen J. Water quality and biofilm formation in dental unit waterline systems in mangaung, South Africa. Int Dent J. (2025) 75(3):2132–49. 10.1016/j.identj.2024.12.025 40023759 PMC12142793

[5] Kohn WG Collins AS Cleveland JL Harte JA Eklund KJ Malvitz DM. Guidelines for infection control in dental health-care settings–2003. MMWR Recomm Rep. (2003) 52(Rr-17):1–61. 14685139

[6] Vinh R Azzolin KA Stream SE Carsten D Eldridge LA Estrich CG. Dental unit waterline infection control practice and knowledge gaps. J Am Dent Assoc. (2024) 155(6):515–525.e1. 10.1016/j.adaj.2024.02.011 38839239

[7] Zhang SG Lu MY Xu LT Zhang Y Wang C Gao R. Monitoring and analysis of bacterial contamination in dental unit waterlines in medical institutions in Nanjing city. Chinese Journal of Disinfection. (2024) 41(01):47–50.

[8] Gawish S Abbass A Abaza A. Occurrence and biofilm forming ability of pseudomonas aeruginosa in the water output of dental unit waterlines in a dental center in alexandria, Egypt. Germs. (2019) 9(2):71–80. 10.18683/germs.2019.1160 31341834 PMC6591527

[9] Khajezadeh M Mohseni F Khaledi A Firoozeh A. Contamination of dental unit water lines (DUWL) with legionella pneumophila and pseudomonas aeruginosa; A Middle East systematic review and meta-analysis. Eur J Microbiol Immunol (Bp). (2023) 12(4):93–9. 10.1556/1886.2022.00023 36626121 PMC9869867

[10] Al-Taee KT Awad FA. Genetic convergence study between pseudomonas aeruginosa isolated from dental units water samples and clinical samples. IMJ. (2020) 25(2):885–90.

[11] Singh J O’Donnell K Nieves DJ Adler-Shohet FC Arrieta AC Ashouri N. Invasive mycobacterium abscessus outbreak at a pediatric dental clinic. Open Forum Infect Dis. (2021) 8(6):ofab165. 10.1093/ofid/ofab165 34113683 PMC8186244

[12] Ricci ML Fontana S Pinci F Fiumana E Pedna MF Farolfi P. Pneumonia associated with a dental unit waterline. Lancet. (2012) 379(9816):684. 10.1016/S0140-6736(12)60074-9 22340301

[13] Samaranayake LP Fakhruddin KS Ngo HC Chang JWW Panduwawala C. The effectiveness and efficacy of respiratory protective equipment (RPE) in dentistry and other health care settings: a systematic review. Acta Odontol Scand. (2020) 78(8):626–39. 10.1080/00016357.2020.1810769 32881590

[14] Association CS. T/CHSA 023-2023: Guidelines for Infection Control and Management in Dental Unit Waterlines. Beijing: Chinese Stomatological Association (2023).

[15] Samaranayake L Fakhruddin K Sobon N Osathanon T. Dental unit waterlines: disinfection and management. Int Dent J. (2024) 74(Suppl 2):S437–45. 10.1016/j.identj.2024.07.1269 39515931 PMC11583879

[16] Fushimi K Watanabe J Yamada M Washio J Takahashi N Egusa H. Effects of flushing of dental waterlines in portable dental units on water quality management. J Dent Sci. (2024) 19(Suppl 1):S61–9. 10.1016/j.jds.2024.10.014 39807432 PMC11725118

[17] Scarano A Murmura G Lorusso F. Efficacy of water filters for dental chair units: assessment of the filtration action versus coxsackievirus B5. J Prev Med Hyg. (2020) 61(2):E296–8. 10.15167/2421-4248/jpmh2020.61.2.1462 32803014 PMC7419115

[18] Marino F Mazzotta M Pascale MR Derelitto C Girolamini L Cristino S. First water safety plan approach applied to a dental clinic complex: identification of new risk factors associated with legionella and P. Aeruginosa contamination, using a novel sampling, maintenance and management program. J Oral Microbiol. (2023) 15(1):2223477. 10.1080/20002297.2023.2223477 37346998 PMC10281406

[19] Mosci D Marmo GW Sciolino L Zaccaro C Antonellini R Accogli L. Automatic environmental disinfection with hydrogen peroxide and silver ions versus manual environmental disinfection with sodium hypochlorite: a multicentre randomized before-and-after trial. J Hosp Infect. (2017) 97(2):175–9. 10.1016/j.jhin.2017.06.010 28610932

[20] Liu ZH Chen YG Jiang N Li Y Lu DM Yang HY. A study of the disinfection effect of sodium hypochlorite on the dental unit waterlines. Chin J Geriatr Dent. (2023) 21(04):247–53.

[21] Yan JX Dang Y Gan L Shuai T Xue SR Li CY. Observation on the disinfection effect of hydrogen peroxide on dental unit waterlines. Chin J Disinfect. (2025) 42(01):4–6.

[22] Offner D Fioretti F Musset AM. Contamination of dental unit waterlines: assessment of three continuous water disinfection systems. BDJ Open. (2016) 2:16007. 10.1038/bdjopen.2016.7 29607068 PMC5842837

[23] ICS Committee. The Basic Protocol-Infection Control Guidelines for the Dental Service. Hong Kong: Department of Health (2019).

[24] Schel AJ Marsh PD Bradshaw DJ Finney M Fulford MR Frandsen E. Comparison of the efficacies of disinfectants to control microbial contamination in dental unit water systems in general dental practices across the European union. Appl Environ Microbiol. (2006) 72(2):1380–7. 10.1128/AEM.72.2.1380-1387.2006 16461690 PMC1392914

[25] Chang J Dang Y Wang CL Li XE. Effect of using hydrogen peroxide for periodic disinfection combined with continuous disinfection to control contamination in dental unit waterline. J Sichuan Univ (Med Sci). (2024) 55(01):217–23. 10.12182/20240160210 PMC10839502 38322511

[26] Shuai T Wen J Liu W Shao T Pei H Li Y. Optimizing hydrogen peroxide shock treatment frequencies for dental unit waterlines contamination control: a pilot study. Bmc Oral Health. (2025) 25(1):1752. 10.1186/s12903-025-06819-0 41194147 PMC12590708

[27] Standardization IOF. ISO 16954:2015: Dentistry-Test Methods for Dental Unit Wastewater Treatment Systems. Geneva: International Organization for Standardization (2015).

[28] Cheng L Naibijiang N Hasenbai A Dong H He H. Bacteriostatic effects of nanometer silver disinfectant on the biofilms in dental unit water lines. J Dent Sci. (2021) 16(1):327–32. 10.1016/j.jds.2020.03.015 33384816 PMC7770243

[29] Ditommaso S Giacomuzzi M Ricciardi E Zotti C. Efficacy of a low dose of hydrogen peroxide (peroxy ag⁺) for continuous treatment of dental unit water lines: challenge test with legionella pneumophila serogroup 1 in a simulated dental unit waterline. Int J Environ Res Public Health. (2016) 13(5):745. 10.3390/ijerph13070745 27455299 PMC4962286

[30] Dang Y Zhang Q Wang J Wang Q Han M Niu Y. Assessment of microbiota diversity in dental unit waterline contamination. PeerJ. (2022) 10:e12723. 10.7717/peerj.12723 35036099 PMC8743008

[31] Kramer A Assadian O Bachfeld D. Purge- and intensive-purge decontamination of dental units contaminated with biofilm. GMS Krankenhhyg Interdiszip. (2012) 7(1):Doc11. 10.3205/dgkh000195 22558045 PMC3334959

[32] Li H Niu YT Sun W Wang CL Li XE Dang Y. Effect of ICX® tablets on controlling the pollution of dental unit waterlines. Chin J Mod Nurs. (2021) 04:508–12.

[33] Pawar A Garg S Mehta S. Breaking the chain of infection: dental unit water quality control. J Clin Diagn Res. (2016) 10(7):Zc80–84. 10.7860/JCDR/2016/19070.8196 27630960 PMC5020288

[34] Zhu C Hong FH Yu X. Anti biofilm effect of low concentration chlorine containing disinfectant assisted by multi enzyme detergent in dental unit waterlines. New Microbiol. (2021) 44(2):117–24. 33978196

[35] Lin S-M Svoboda KKH Giletto A Seibert J Puttaiah R. Effects of hydrogen peroxide on dental unit biofilms and treatment water contamination. Eur J Dent. (2011) 05(1):047–59. 10.1055/s-0039-1698858 PMC3019751 21228956

[36] Baudet A Lizon J Martrette J-M Camelot F Florentin A Clément C. Efficacy of BRS(®) and alpron(®)/bilpron(®) disinfectants for dental unit waterlines: a six-year study. Int J Environ Res Public Health. (2020) 17(8):2634. 10.3390/ijerph17082634 32290533 PMC7215904

